# Imaging findings of vitamin deficiencies: are they forgotten diseases?

**DOI:** 10.1259/bjro.20210011

**Published:** 2021-04-04

**Authors:** Akitoshi Inoue, Kentaro Itabashi, Takayasu Iwai, Hitoshi Kitahara, Yoshiyuki Watanabe

**Affiliations:** ^1^ Department of Radiology, Shiga University of Medical Science, Shiga, Japan; ^2^ Department of Radiology, Kohka Public Hospital, Shiga, Japan; ^3^ Department of Radiology, Omihachiman Community Medical Center, Shiga, Japan

## Abstract

Vitamin deficiency is rare in modern industrialised countries; however, it still occurs in patients with specific backgrounds, such as those with extremely unbalanced diets, those with alcoholism and those who have undergone gastrointestinal surgery. Imaging examinations that demonstrate classic findings confirm the clinical diagnosis of vitamin deficiency and help monitor response to treatment. Because vitamin deficiencies are not prevalent, the diagnosis might not be straightforward. Therefore, imaging should be performed in cases of suspected vitamin deficiency. Radiologists should be familiar with characteristic imaging findings of vitamin deficiency and should survey an affected patient’s background and blood vitamin levels. Because symptoms of vitamin deficiency are quickly improved by vitamin replacement, early diagnosis is essential. This pictorial review provides imaging findings for deficiencies in vitamins B1 (Wernicke encephalopathy and wet beriberi), B12 (subacute combined degeneration), C (scurvy), D (rickets) and K (bleeding tendency).

## Introduction

In modern industrialised countries, vitamin deficiency is rare because the food supply is stable and sufficient. Vitamin deficiency may result from inadequate oral intake or malabsorption. Extreme picky eaters, such as people with autism spectrum disorder or schizophrenia and patients with alcoholism, may develop symptoms of vitamin deficiency. A history of surgery in the gastrointestinal tract is also a risk factor.

Physicians might not be familiar with vitamin deficiency because its symptoms are relatively mild; therefore, the diagnosis may be difficult and delayed. Although vitamin deficiency is basically diagnosed through medical interviews and physical examinations, some vitamin deficiencies are characterised by specific imaging findings, which may aid in the diagnosis. The symptoms of vitamin deficiencies are quickly resolved after sufficient vitamin supplementation; therefore, knowledge of imaging findings of vitamin deficiency helps improve patient outcomes. This pictorial essay provides imaging findings of vitamin deficiencies (Table 1).

**Table 1.** Summary of clinical manifestation and imaging findings of vitamin deficiencies

	Clinical manifestation	Typical imaging findings
**Vitamin B1**	**Wernicke encephalopathy**	
	Altered mental status Oculomotor disorder Ataxia	Symmetrical hyperintensity lesions in paraventricular regions of the thalamus, hypothalamus, mamillary bodies, periaqueductal region and floor of the fourth ventricle on *T*_2_WI, FLAIR and DWI
	**Wet beriberi**	
	High-output heart failure	Cardiomegaly Pericardial effusion (high density on non-contrast enhanced CT) Myocardial oedema on *T*_2_WI
**Vitamin B12**	**Subacute combined degeneration**	
	Symmetrical weakness, paraesthesia, sensory ataxia and loss of vibratory sense	Symmetrical high-intensity area in the posterior and lateral column on *T*_2_WI (inverted V sign, dot sign, three-point sign)
**Vitamin C**	**Scurvy**	
	Leg pain Gait abnormality Inability to walk Delayed wound healing Nail change Corkscrew hair Gingival and tooth anomalies Anaemia	**Endochondral ossification impairment (children**) Frankel line, Trummerfeld zone, Pelkan spurs, Wimberger ring sign on radiographs **Bone turnover failure** Disuse osteopaenia, thin cortices on radiograph **Bleeding tendency** Subosteal haemorrhage on ultrasonography and MRI Intramuscular haemorrhages with branching pattern **Other** Patchy or diffuse abnormal intensity or enhancement in the bone marrow on MRI Hyperintensity in the muscle on MRI Subcutaneous and fascial oedema Hot spots in femoral, tibial and fibular metaphyses on bone scan
**Vitamin D**	**Rickets**	
	Delayed growth Delayed motor skills Pain in the spine, pelvis, legs and ribs	Widening of the growth plate Cupping, splaying and fraying of the metaphyses
	**Osteomalacia**	
	Pain in the spine, pelvis, legs and ribs	Decreased bone mass Looser zone (insufficient fracture without traumatic episode)
**Vitamin K**	**Infantile intracranial haemorrhage**	
	Lethargy, inactivity	Intraparenchymal haemorrhage, subarachnoid haemorrhage, subdural haematoma
	**Bleeding tendency**	
	Purpura Gastrointestinal bleeding Epistaxis Haematuria	Depends on the involved organ

DWI, diffusion-weighted imaging; FLAIR, fluid-attenuated inversion recovery; *T*_2_WI, *T*_2_ weighted imaging.

### Vitamin B1 deficiency

Vitamin B1 deficiency occurs predominantly in patients who have undergone gastrointestinal surgery and in patients with alcoholism because vitamin B1 (thiamine) is absorbed in the jejunum, and because ethanol inhibits the absorption of vitamin B1.

**Figure 1.**
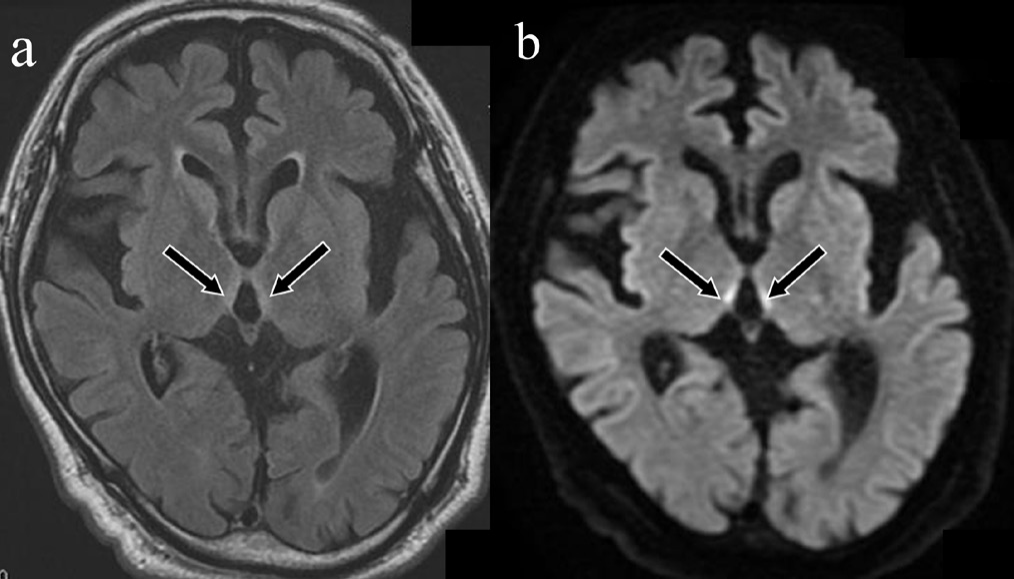
Wernicke encephalopathy in alcoholism. A 54-year-old male was found lying on the ground. He exhibited severe disturbance of consciousness, and his Glasgow Coma Scale score was 3. He was hospitalised to manage his alcoholism. Laboratory tests revealed a decreased vitamin B1 level (23 ng ml^−1^ [normal range: 24–66 ng ml^−1^]). The symmetrical hyperintensity lesions around the third ventricle were observed on fluid-attenuated inversion recovery imaging (a: arrows). These lesions were more conspicuous on diffusion-weighted imaging (b: arrows).

**Figure 2.**
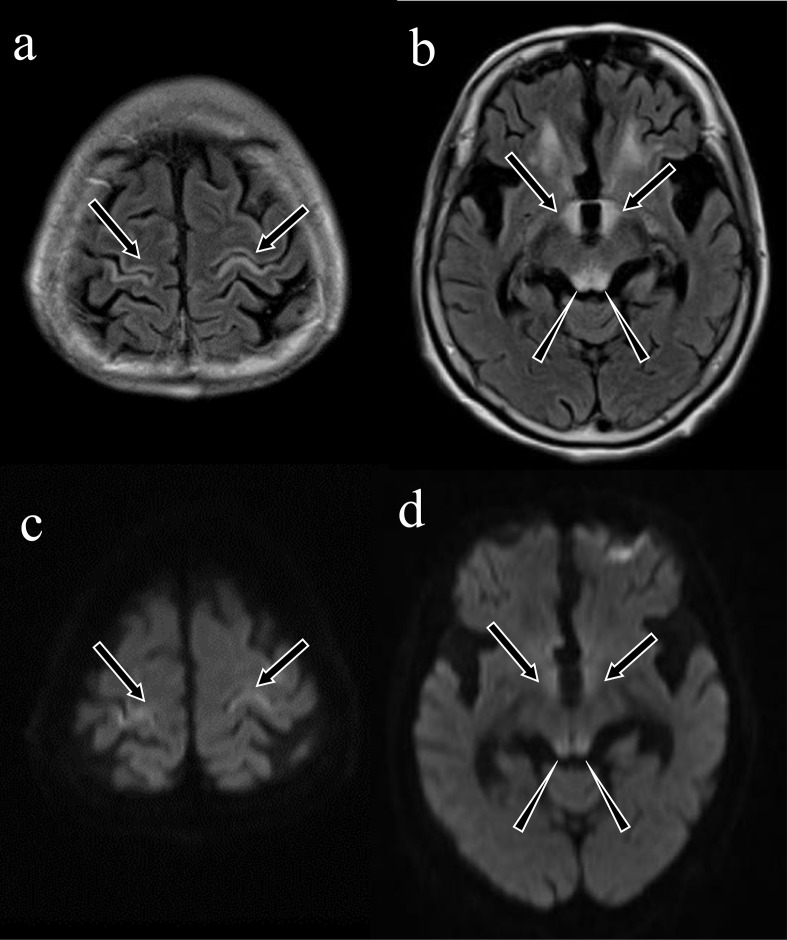
Wernicke encephalopathy with cortical involvement. An 82-year-old female who received a large infusion as a treatment for acute pancreatitis and hyponatraemia demonstrated disturbance of consciousness. Osmotic encephalopathy was suspected because of the hyponatraemia and infusion therapy. Fluid-attenuated inversion recovery imaging (**a, b**) and diffusion-weighted imaging (**c, d**) revealed symmetrical lesions of hyperintensity in the bilateral postcentral gyrus (a, c: arrows), corpora quadrigemina (b, d: arrowheads) and brain parenchyma around the third ventricle (b, d: arrows), which are highly suggestive of Wernicke encephalopathy. Her vitamin B1 level was decreased (12 ng ml^−1^ [normal range: 24–66 ng ml^−1^]).

Wernicke encephalopathy is an acute neurological disorder caused by vitamin B1 deficiency. The classical triad of clinical symptoms—altered mental status, ataxia and oculomotor disorder—is observed in only 16–33% of patients; a late development is a severe neurological disorder known as Korsakoff psychosis charactarized by anterograde and retrograde amnesia.^1^ Lesions adjacent to the ventricles are better detected on MRI, fluid-attenuated inversion recovery (FLAIR) sequences and diffusion-weighted imaging (DWI) than on *T*_2_ weighted imaging (*T*_2_WI). Common locations of lesions are symmetrical in the paraventricular regions of the thalamus, hypothalamus, mammillary bodies, periaqueductal region and floor of the fourth ventricle (Figure 1), whereas uncommon locations include the putamen, caudate, splenium of the corpus callosum, dorsal medulla, pons, red nucleus, substantia nigra of the midbrain, vermis, dentate nucleus, paravermis, fornix and areas of the cerebral cortex, such as pre- and post-central gyri (Figure 2).^1^ Cortical involvement suggests irreversible damage and an unfavourable prognosis.^2^

**Figure 3.**
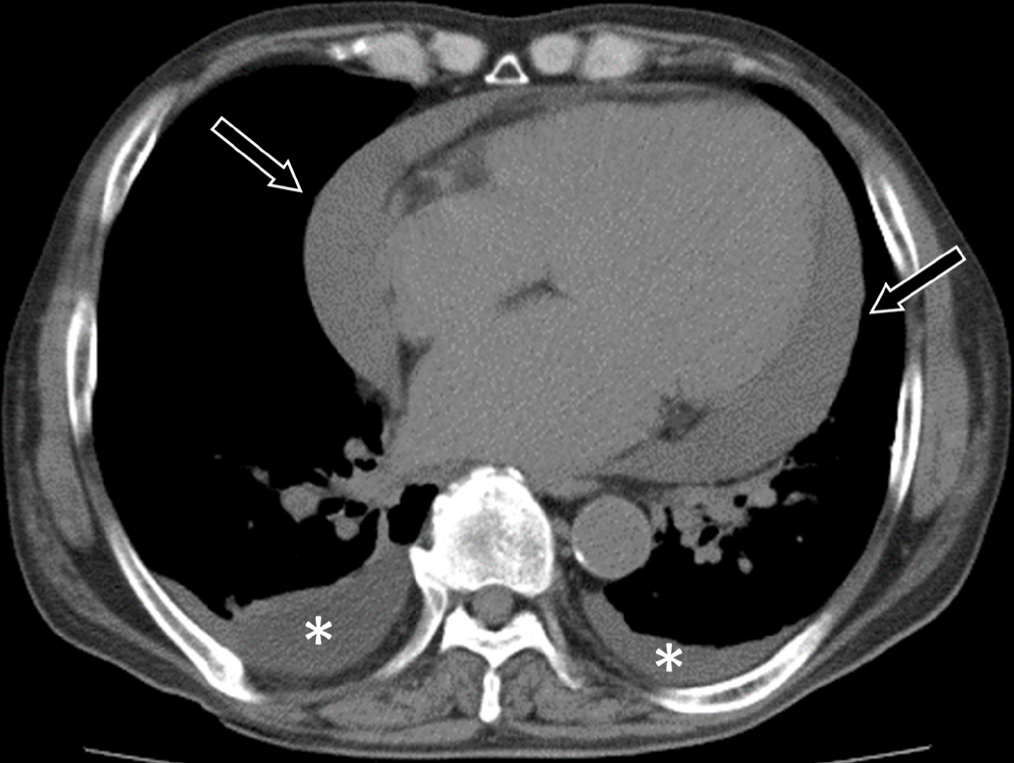
Wet beriberi. A 70-year-old male presented with dyspnoea, nausea and appetite loss in the emergency room. He presented with normal blood pressure (101/71 mmHg) but tachycardia (heart rate: 107 bpm). Arterial blood gas analysis revealed acidosis (pH: 7.256 [normal range: 7.36–7.44]), a decreased pressure of CO2 (10 mm Hg [normal range: 35–45 mm Hg]), a decreased base excess (−20 mEq/L [normal range: −2—+2 mEq l^−1^ ]), an increased lactate level (12 mmol l^−1^ [normal range:<5 mmol l^−1^]), and an unremarkable arterial partial pressure of oxygen (104 mm Hg [normal range:>80 mm Hg]), which means metabolic acidosis due to elevated lactic level. His cardiac index was 3.0, and his pulmonary capillary wedge pressure was 23 mm Hg, which signified Forrester Class II heart failure. Laboratory tests revealed a decreased vitamin B1 level (17 ng ml^−1^ [normal range: 24–66 ng ml^−1^]). CT demonstrated bilateral pleural effusion (asterisks), cardiomegaly and a collection of pericardial fluid effusion that was more highly attenuated than pleural effusion (arrows).

Beriberi is the other phenotype caused by vitamin B1 deficiency. Wet beriberi is characterised by high-output heart failure and low peripheral vascular resistance, whereas dry beriberi is a peripheral nerve disorder. In addition to the examination of a patient’s medical background, wet beriberi is usually diagnosed through a combination of arterial blood gas analysis, echocardiography and cardiac catheterisation. The imaging findings of wet beriberi are not well-known. Patients with wet beriberi may demonstrate exudative pericardial effusion. In an earlier article, investigators speculated that increased hydrostatic pressure causes pericardial effusion; however, the mechanism has not been elucidated.^3^ The CT number of the pericardial effusion may be higher than that of pure water because of the exudative nature of the effusion (Figure 3). T2 mapping demonstrated reversible elevated myocardial T2 values, suggestive of diffuse oedema,^4^ which is compatible with a reported histopathological feature: colliquative myocytolysis, defined as progressive loss of myofibrils in conjunction with intramyocellular oedema.^5^ Wet beriberi sometimes co-exists with Wernicke encephalopathy.

### Vitamin B12 deficiency

**Figure 4.**
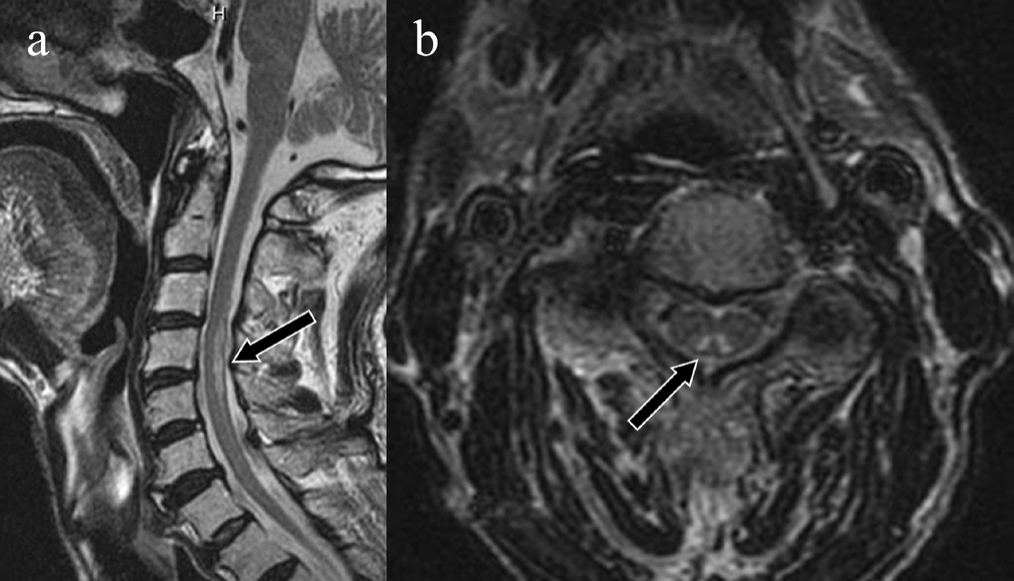
Subacute combined degeneration of the spinal cord. A 72-year-old male who had undergone total gastrectomy for gastric cancer 11 years earlier presented with bilateral numbness in the upper extremities. Sagittal *T*_2_WI showed a high-intensity lesion at the level of C4 to C5 (a: arrow), and axial *T*_2_WI showed a symmetrical high-intensity lesion in the posterior column, known as the ‘inverted V’ sign (b: arrow). His vitamin B12 level (130 pg ml^−1^) was lower than the normal range (249–938 pg ml^−1^). *T*_2_WI, *T*_2_ weighted imaging.

Vitamin B12 (cobalamin) originates from animal food and is synthesised by a microorganism. Absorption in the distal ileum depends on intrinsic factor, which is synthesised by the gastric parietal cells; therefore, total gastrectomy is a risk factor for vitamin B12 deficiency. This disorder leads to megaloblastic anaemia, which may result from folate deficiency and subacute combined degeneration. The symptoms of subacute combined degeneration include symmetrical weakness, paraesthesia, sensory ataxia and loss of vibratory sense with subacute onset and progression from distal to proximal. On MRI, a characteristic finding is symmetrical high T2 signal in the posterior and lateral columns, involving the corticospinal and spinocerebellar tracts (Figure 4). The characteristic findings on axial *T*_2_WI have been described as an ‘inverted V sign’, a ‘dot sign’ or a ‘three-point sign’.^6^ Pathological findings are characterised by cord demyelination with axonal loss. Enhancement depends on the presence of damage to the blood–spine barrier.^7^

### Vitamin C deficiency

Vitamin C (ascorbic acid) is obtained from fruits and vegetables and is absorbed in the ileum. It functions as an antioxidant and a cofactor for the hydroxylation of protein and is essential in synthesising collagen. Vitamin C deficiency, known as scurvy, leads to abnormal collagen production. Because collagen is distributed in every part of the body, the various manifestations of scurvy include delayed wound healing, nail change, corkscrew hair, gingival and tooth anomalies and anaemia, which sometimes mimics haematological disease, vasculitis and infection. Blood vessels become fragile as a result of abnormal collagen, and this can lead to a bleeding tendency. In affected children, subperiosteal haemorrhage as a result of bleeding tendency can cause inability to walk and leg pain, especially around the knee.^8^

**Figure 5.**
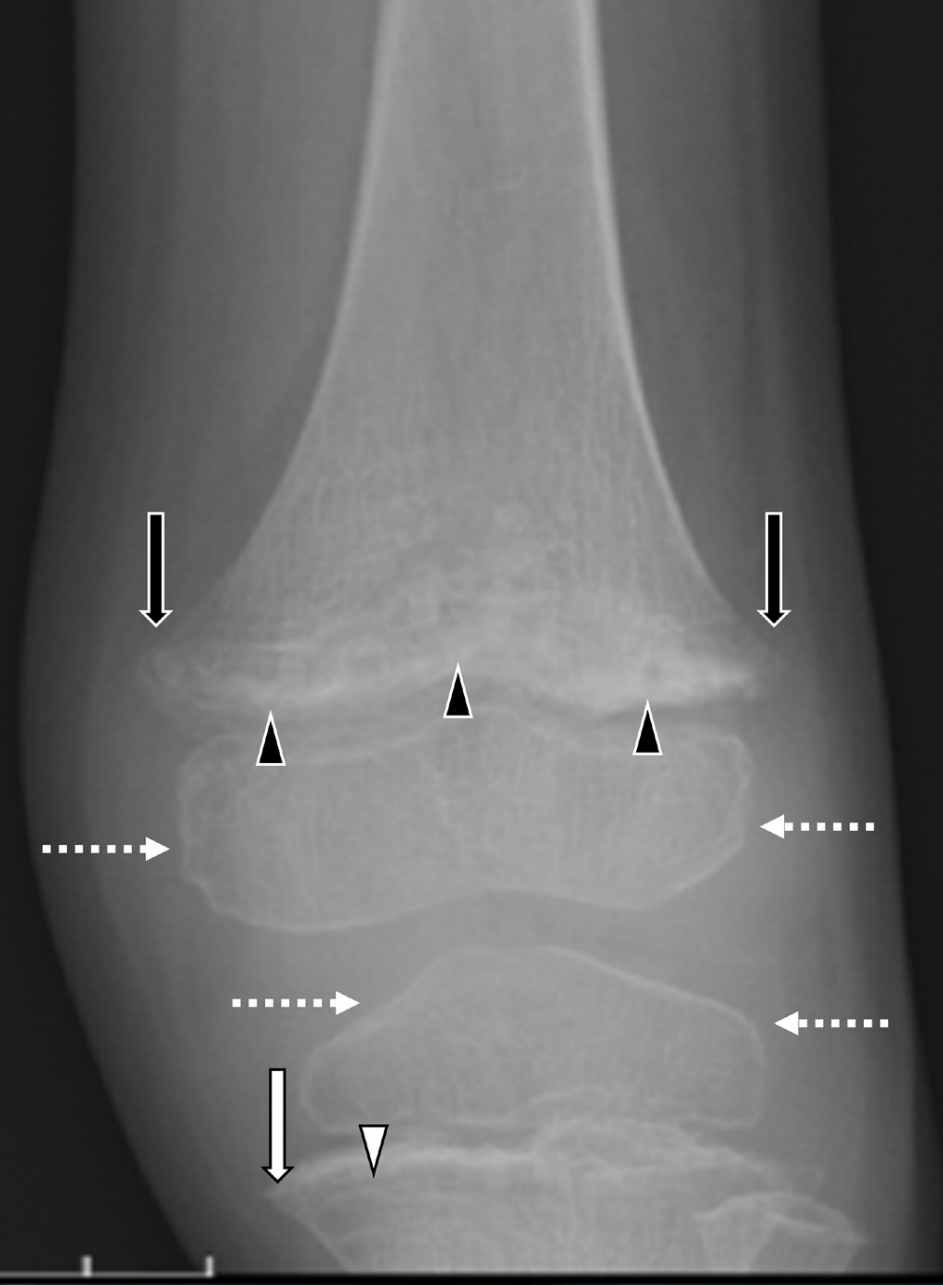
Paediatric case of scurvy demonstrated on radiography. A 4-year-old boy with autistic spectrum disorder suffered from left thigh pain. Anteroposterior radiographs demonstrated the heterogeneous and irregular appearance of the growth plate (black arrowheads) and metaphyseal beaking (Pelkan spur: black arrows) in the femur and a sclerotic cortical rim around osteopaenic epiphyseal ossification centres of the femur and tibia (Wimberger ring sign: broken white arrows). In the tibia, a dense metaphyseal band (Frankel line; arrow) and a lucent band (scurvy line; white arrowhead) are visible. Laboratory examination revealed lower vitamin C level (0.2 µg ml^−1^ [range: 4.7—17.8 µg ml^−1^]).

**Figure 6.**
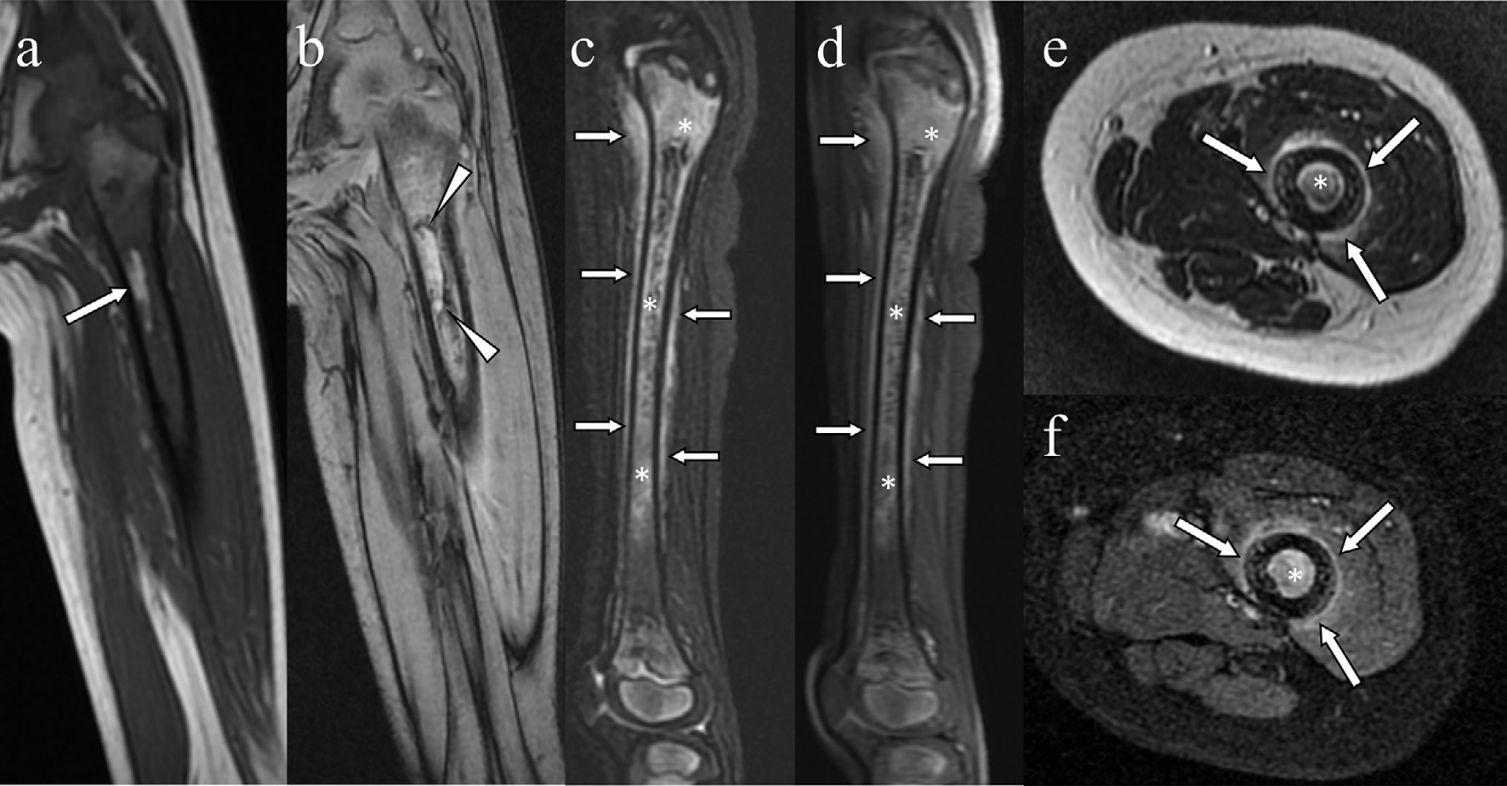
Paediatric case of scurvy (same case as in Figure 5). A spotty hyperintensity lesion in the diffuse hypointense bone marrow of the femur was visible on coronal *T*_1_ weighted magnetic resonance imaging (a: arrow). The rim-like hypointensity around the lesion (arrowheads) was suggestive of haemosiderin around the bone marrow lesion on coronal *T*_2_* weighted imaging (b: arrowheads). The bone marrow was diffusely hyperintense and contained a heterogeneous hypointense area (asterisks) in association with hyperintensity along the periosteum (arrows), as observed on sagittal fat-suppressed *T*_2_ weighted imaging (**c**) and contrast enhanced fat suppressed *T*_1_ weighted imaging (**d**). The contrast enhancements with hyperintensity were seen in the bone marrow and periosteum on axial *T*_2_ weighted imaging (e: arrows) and on contrast-enhanced fat-suppressed *T*_1_ weighted imaging (f: arrows).

The musculoskeletal abnormality may be categorised as impairment in endochondral ossification; bone turnover failure, which leads to abnormal development of bone matrix, and a bleeding tendency. The manifestations of endochondral ossification impairment are limited in paediatric patients. Musculoskeletal radiographic findings of paediatric scurvy have been well documented. Scurvy may involve the distal femur, proximal tibia and fibula, distal radius and ulna, proximal humerus and distal rib ends. Radiographic findings include a dense band along the metaphyseal side of the growth plate in the provisional zone calcification (Frankel line) with subjacent lines of demineralisation (scurvy line or Trummerfeld zone), a peripheral extension of the zone of calcification that results in beaking (Pelkan spurs) and a sclerotic cortical rim around osteopenic epiphyseal ossification centres (Wimberger ring sign) (Figure 5).^9^ Moreover, diffuse osteopaenia and thin cortices distributed predominantly in the epiphyses are observed on radiographs. On nuclear bone scans, hot spots are observed in femoral, tibial and fibular metaphyses corresponded to a provisional zone calcification (Frankel line).^10^ Ultrasonography reveals subperiosteal heterogenous echogenic effusion, suggestive of subperiosteal haemorrhage.^11^ MRI can also depict subperiosteal haemorrhage, as well as bone marrow abnormalities, which demonstrate diffuse or patchy abnormal signal intensity; the characteristic findings are symmetrical low intensity on *T*_1_ weighted images, high-intensity on fat-suppressed *T*_2_ weighted images and contrast enhancement on fat-suppressed *T*_1_ weighted images in the bilateral lower extremity metaphyses^12^ (Figure 6). Because this bone marrow abnormality may mimic osteomyelitis, leukaemia and primary bone tumour, an unnecessary bone biopsy is sometimes performed to determine malignancy pathologically. The bone marrow abnormality corresponds to gelatinous transformation caused by the accumulation of acid mucopolysaccharides.^13^

**Figure 7.**
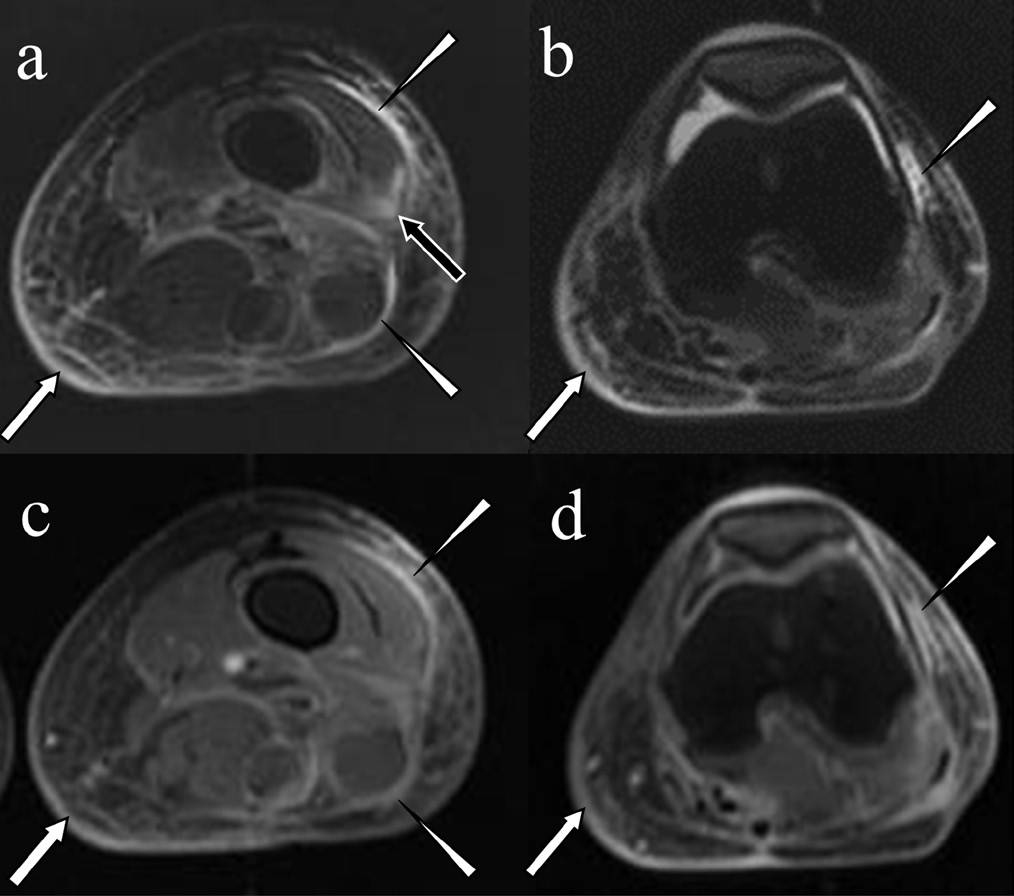
Adult case of scurvy. A 40-year-old female who had schizophrenia, a manifestation of which was an unbalanced diet, presented with a swollen left lower extremity with bleeding spots. Fat-suppressed *T*_2_ weighted images demonstrated linear hyperintensity on the fascia (a; arrowheads) and the lateral retinaculum of the patella (b; arrowhead), an area of intramuscular hyperintensity (a; black arrow) and thickened skin in association with reticular hyperintensity in the subcutaneous tissue (a and b; white arrows). On fat-suppressed *T*_1_ weighted imaging after intravenous gadolinium injection, contrast enhancement was observed in the fascia (c; arrowheads) and the lateral retinaculum of the patella (d; arrowhead) and thickened skin (c and d; arrows). Laboratory examination demonstrated an extremely low vitamin C level (0.6 µg ml^−1^ [normal range: 4.7—17.8 µg ml^−1^]).

Reports of adult scurvy are limited. Multiple small bilateral intramuscular haemorrhages with a branching pattern suggestive of perivascular distribution were reported as a unique presentation of scurvy.^14^ Another case report described nonspecific MRI depictions of inflammation, including dermal thickening, subcutaneous and deep fascial oedema and heterogeneous increased *T*_2_ weighted signal bilaterally in the quadriceps and gastrocnemius muscles (Figure 7). The mechanism of contrast enhancement and the hyperintensity of lesions on *T*_2_WI are believed to reflect increased capillary permeability as a result of the fragility of the vessel wall. In one report, patchy foci of enhancing marrow oedema in the distal femur and proximal tibia were non-specific and assumed to represent islands of red marrow.^15^

### Vitamin D deficiency

Vitamin D is obtained through exposure to sunlight or diet. Activated vitamin D is involved in calcium, phosphorus and bone metabolism; therefore, its deficiency results in rickets, which is the interruption of development and mineralisation of the growth plates, or osteomalacia, which is insufficient or abnormal mineralisation of osteoid in the bone. Rickets occurs only before the growth plate fusion.^16^

**Figure 8.**
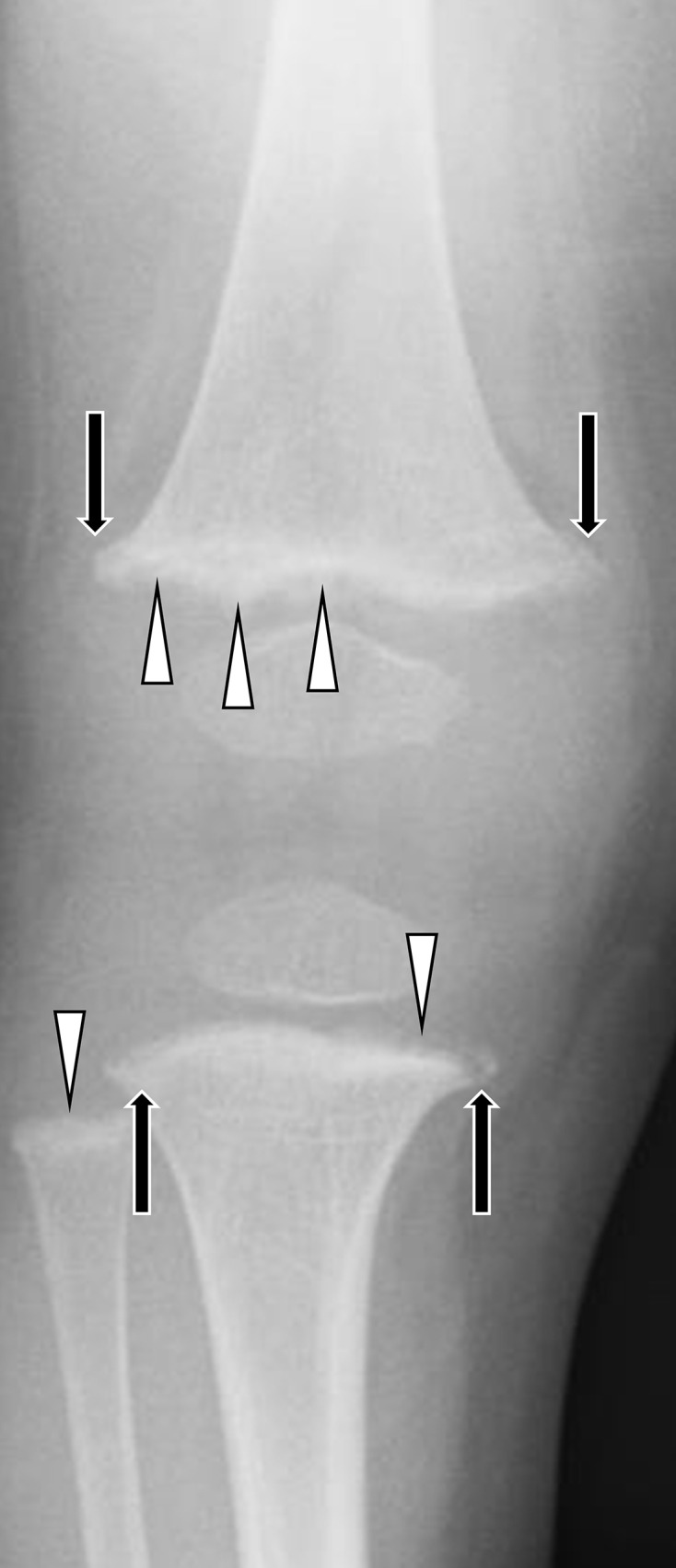
Rickets in the knee. An 18-month-old boy who was exclusively breastfed lost his appetite after starting nursery school. His mother was aware of the difference of the length of his lower extremities. Posteroanterior radiograph demonstrated indistinct metaphyseal margins in the femur, tibia and fibula (fraying; arrowheads) and the widening of metaphyseal ends in the femur and tibia (splaying; black arrows). Healing stage was suggested due to the provisional zone of calcification although actual onset was unknown. His activated vitamin D level (0.87 pg ml^−1^) was lower than the paediatric normal range (20–70 pg ml^−1^).

**Figure 9.**
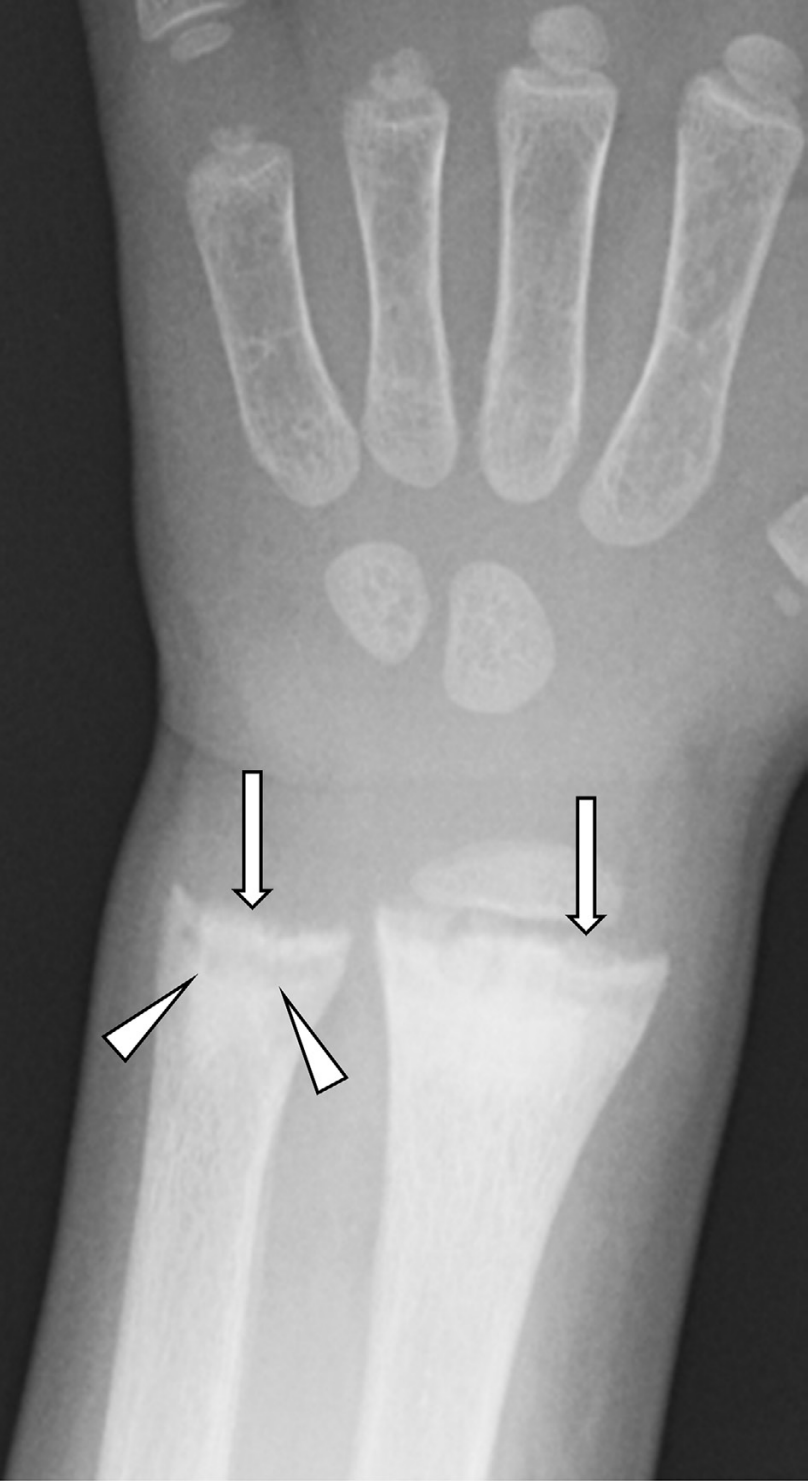
Rickets in the wrist. A 3-year-old boy who was an extremely picky eater and had a cognitive delay. He always stayed at home and rarely played outside. Anteroposterior radiograph of the left wrist showed concave deformity of the growth plate of the ulna (cupping: arrowheads) and an indistinct metaphyseal margin (fraying: arrows) in the radius and ulna. His activated vitamin D level (<5 pg ml^−1^) was lower than the paediatric normal range (20–70 pg ml^−1^).

Patients with rickets may present with pain and exhibit delayed growth and motor skills. On radiographs, the characteristic findings are widening of the growth plate; cupping, splaying and fraying of the metaphyses; and poor mineralisation (Figures 8 and 9). Transverse lucent bands (Looser zones), which are pseudofractures caused by no particular traumatic episode, are identified on radiographs. Osteomalacia may be asymptomatic in the early stage; however, as it progresses, affected patients may have pain in the lower back, pelvis, hips, legs and ribs. Decreased bone mass and Looser zones are findings of osteomalacia. The most common sites are the inner margin of the femoral neck and the pubic rami but can be observed in other sites.^9^

### Vitamin K deficiency

**Figure 10.**
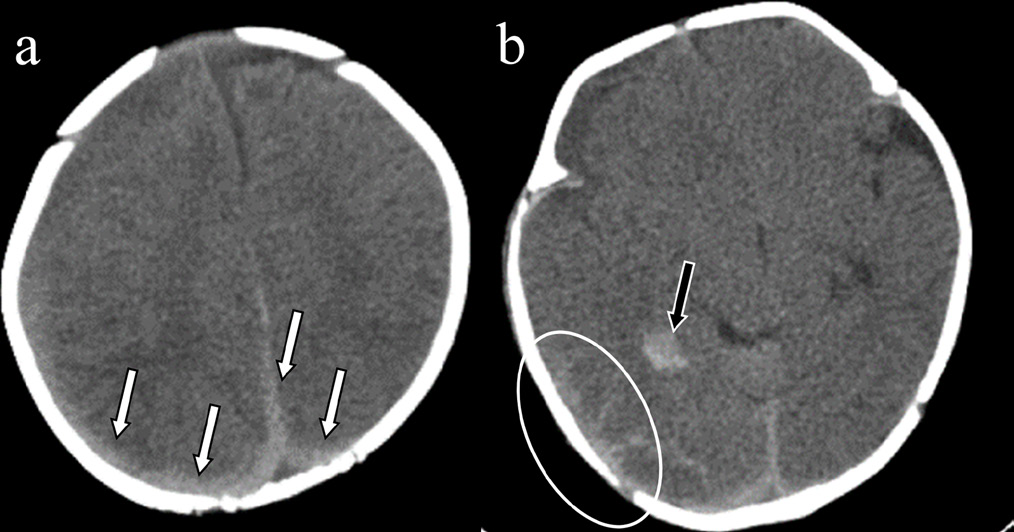
Intracranial haemorrhage caused by vitamin K deficiency. A 2-month-old boy presented with disturbance of consciousness and decreased feeding. Head computed tomography demonstrated subdural haematoma around the falx and posterior area (a; white arrows), subarachnoid haemorrhage on the surface of the right temporal lobe (b; circle) and intraparenchymal haemorrhage in the temporal lobe (b; black arrow). He was examined the causes of bleeding tendency and diagnosed with biliary atresia.

Vitamin K is indispensable for synthesising coagulation factors II, VII, IX and X, which are essential for haemostasis. Vitamin K deficiency is common in breastfed infants because human breast milk contains a low amount of vitamin K; additionally, stores of vitamin K are low at birth. Biliary atresia is associated with vitamin K deficiency because vitamin K is fat-soluble and bile is needed for its absorption. Intracranial haemorrhage is one of the life-threatening manifestations of vitamin K deficiency in infants (Figure 10), although jaundice and acholic stools are common initial manifestations in children with biliary atresia.^17^ In the adult population, vitamin K deficiency is rare because the daily requirement is small. However, an extremely unbalanced diet may cause bleeding tendency, purpura, gastrointestinal bleeding, epistaxis and haematuria.

## Conclusion

Vitamin deficiencies can develop in patients with an extremely unbalanced diet, those who have undergone gastrointestinal surgery or those with mental disease even in modern industrialised countries. Because physician might be not aware of vitamin deficiencies due to its low prevalence, radiologists should be familiar with characteristic imaging findings of vitamin deficiencies for correct and early diagnosis and treatment.
